# Trends in Adaptive Design Methods in Dialysis Clinical Trials: A Systematic Review

**DOI:** 10.1016/j.xkme.2021.08.001

**Published:** 2021-08-20

**Authors:** Conor Judge, Robert Murphy, Catriona Reddin, Sarah Cormican, Andrew Smyth, Martin O’Halloran, Martin J. O’Donnell

**Affiliations:** 1 HRB-Clinical Research Facility Galway, NUI Galway, Galway, Ireland; 2 Translational Medical Device Lab, NUI Galway, Galway, Ireland; 3 Wellcome Trust-HRB, Irish Clinical Academic Training, Dublin, Ireland

**Keywords:** Dialysis, adaptive design, hemodialysis, peritoneal dialysis, end stage kidney disease

## Abstract

**Rationale & Objective:**

Adaptive design methods are intended to improve the efficiency of clinical trials and are relevant to evaluating interventions in dialysis populations. We sought to determine the use of adaptive designs in dialysis clinical trials and quantify trends in their use over time.

**Study Design:**

We completed a novel full-text systematic review that used a machine learning classifier (RobotSearch) for filtering randomized controlled trials and adhered to the Preferred Reporting Items for Systematic Review and Meta-analysis (PRISMA) guidelines.

**Setting & Study Populations:**

We searched MEDLINE (PubMed) and ClinicalTrials.gov using sensitive dialysis search terms.

**Selection Criteria for Studies:**

We included all randomized clinical trials with patients receiving dialysis or clinical trials with dialysis as a primary or secondary outcome. There was no restriction of disease type or intervention type.

**Data Extraction & Analytical Approach:**

We performed a detailed data extraction of trial characteristics and a completed a narrative synthesis of the data.

**Results:**

57 studies, available as 68 articles and 7 ClinicalTrials.gov summaries, were included after full-text review (initial search, 209,033 PubMed abstracts and 6,002 ClinicalTrials.gov summaries). 31 studies were conducted in a dialysis population and 26 studies included dialysis as a primary or secondary outcome. Although the absolute number of adaptive design methods is increasing over time, the relative use of adaptive design methods in dialysis trials is decreasing over time (6.12% in 2009 to 0.43% in 2019, with a mean of 1.82%). Group sequential designs were the most common type of adaptive design method used. Adaptive design methods affected the conduct of 50.9% of trials, most commonly resulting in stopping early for futility (41.2%) and early stopping for safety (23.5%). Acute kidney injury was studied in 32 trials (56.1%), kidney failure requiring dialysis was studied in 24 trials (42.1%), and chronic kidney disease was studied in 1 trial (1.75%). 27 studies (47.4%) were supported by public funding. 44 studies (77.2%) did not report their adaptive design method in the title or abstract and would not be detected by a standard systematic review.

**Limitations:**

We limited our search to 2 databases (PubMed and ClinicalTrials.gov) due to the scale of studies sourced (209,033 and 6,002 results, respectively).

**Conclusions:**

Adaptive design methods are used in dialysis trials but there has been a decline in their relative use over time.

**Plain-Language Summary**

Adaptive designs make clinical trials more efficient and are one part of the solution for optimizing the design of clinical trials in dialysis. We performed a systematic review by searching 2 large databases for dialysis trials with adaptive designs and found 57 examples. They are used mostly in trials of acute kidney injury, affected (changed a trial) half the studies they were used in, and are usually not reported in titles or abstracts of articles. We also found that the relative use of adaptive designs in nephrology is decreasing over time. Greater knowledge of adaptive design examples in dialysis will further improve uptake in dialysis randomized clinical trials.

Randomized clinical trials (RCTs) are the gold standard for evaluating the efficacy, futility, or harm of new therapies.^2^ Compared with similar medical specialties, nephrology has traditionally had a low number of RCTs, particularly evident for patients with kidney failure requiring dialysis.^3^ The comparatively low number of trials are postulated to be due to difficult recruitment, previous history of underpowered trials, and lack of funding.^4^^,^^5^ Although the number of trials is increasing, nephrology continues to lag behind other specialties such as cardiology, hematology/oncology, and gastroenterology.^6^^,^^7^^,^∗

Adaptive clinical trials use interim data analyses to modify the trial design or duration in a predefined way^8^ without undermining the integrity or validity of the trial, thereby preserving the type 1 error (false-positive) rate. The most common type of adaptive design is the group sequential design, in which planned interim analyses permit stopping of trials for efficacy or futility. Other designs include sample size re-estimation, multiarm multistage trials, adaptive randomization, biomarker adaptive, and seamless phase 2/3 trials^9^ (Box 1).

Box 1 **Adaptive Trial Designs**

**Seamless phase 2-3 design:** Combines a traditional phase 2 with a phase 3 trial. Referred to as the “learning” phase and “confirmatory” phase. This design can reduce sample size and time to market for a positive treatment.

**Sample-size re-estimation design:** Allows for sample-size adjustment or re-estimation based on the results of interim analysis. Particularly useful if there is uncertainty about the treatment effect and variability and when inaccurate estimates could lead to overpowered or underpowered trials.

**GSD:** Allows a trial to stop early based on the results of interim analysis. GSD is the most common type of adaptive design. GSD can take 3 forms: early efficacy stopping, early futility stopping, and early efficacy or futility stopping design.

**Multiarm multistage:** A multistage design with several treatment arms. At interim analysis, inferior treatment arms are dropped based on prespecified criteria. Ultimately the best arms and the control group are retained. Some examples are pick-the-winners or drop-the-loser designs.

**Biomarker-adaptive design:** Allows for adaptations using information obtained from biomarkers. Often used in drug trials to target very selective populations for whom the drug likely works well. The biomarker response at interim analysis can be used to determine the target population.

**Adaptive dose-escalation design:** The dose level used to treat the next patient is based on the toxicity in the previous patients and escalation rules.

Abbreviation: GSD, group sequential design,

Adaptive clinical trials appear particularly suitable for the evaluation of novel interventions in dialysis by reducing resource requirements, decreasing time to study completion, and increasing the likelihood of study success, that is, power to answer hypothesis.^10^ Previous trials in dialysis have overly relied on observational data to inform trial design, including assumptions of expected effect size and variance,^11^ rather than estimates from early-phase clinical trials. If incorrect, trials may be underpowered with an insufficient sample size to answer the underlying research question.^11^ Adaptive sample size re-estimation is a potential solution, as commonly used in cardiology trials,^12^ such as planned blinded sample size re-estimation, which identifies inaccurate assumptions, thereby triggering altered recruitment targets midtrial to ensure adequate power.

Adaptive design may also be relevant when evaluating more established interventions. For example, the Deutsche Diabetes Dialyse Studie (4D)^13^ reported that atorvastatin, 20 mg per day, did not reduce cardiovascular events in kidney failure requiring dialysis despite evidence of a 20% to 30% reduction in other populations.^14^ This trial included a single dose of statin; it is hypothesized that alternative or multiple doses may have been more beneficial in a dialysis population given the significantly altered pharmacokinetics and pharmacodynamics.^11^^,^^15^ An adaptive multiarm multistage trial design may have been more appropriate with 1 interim analysis at the end of stage I to identify an optimum dose to take forward into stage II. For example, the Telmisartan and Insulin Resistance in HIV (TAILoR) trial used a multiarm multistage design with 1 interim analysis to identify the most appropriate dose among 3 telmisartan doses (20, 40, and 80 mg daily). All 3 doses were tested in stage I and telmisartan, 80 mg, was taken forward into stage II.^16^

This systematic review aims to: (1) summarize the use of adaptive design methodology in RCTs in dialysis populations and populations at risk for requiring dialysis; (2) describe the characteristics of the trials that use adaptive designs, including dialysis modality, funding, and geographical location; (3) describe the characteristics of adaptive trial designs in dialysis trials; (4) estimate the percentage of adaptive clinical trials in dialysis among all dialysis RCT; and (5) outline temporal trends in all of the above.

## Methods

We performed a systematic review, reported according to the Preferred Reporting Items for Systematic Reviews and Meta-analyses (PRISMA) guidelines.^17^ The protocol was registered with PROSPERO (CRD42020163946) and published separately.^18^ There were no age or English language restrictions. After testing our predefined search strategy,^18^ we found a small number (n = 16) of dialysis RCTs that reported an adaptive design method. We discovered that the adaptive design methods are often not reported in the title and abstract of articles and would not be detected in a traditional systematic search. To overcome this, we developed a novel “full-text systematic review” protocol and to our knowledge, this is the first use of this methodology.

### Search Method for the Identification of Trials

#### Electronic Search: Dialysis Studies

We performed an electronic search on MEDLINE (PubMed) and ClinicalTrials.gov from database inception until June 1, 2020. Zotero was used as our reference manager. The dialysis search terms were adapted from Beaubien-Souligny et al,^19^ 2019 (and included dialysis, peritoneal dialysis, hemodialysis, hemodiafiltration, hemodiafiltration, hemofiltration, haemofiltration, extracorporeal blood cleansing, haemodialysis, renal dialysis, renal replacement, end stage kidney, end stage renal, stage 5 kidney, and stage 5 renal (Table S1). The output was stored in the Research Information Systems file format for PubMed and XML files for ClinicalTrials.gov.

#### Machine Learning Classifier: RCTs

We used the high-sensitivity machine learning classifier (RobotSearch) to identify RCTs from the PubMed dialysis search output.^15^ RobotSearch is a machine learning classification algorithm combining an ensemble of support vector machines and convolutional neural networks with a reported area under the curve of 0.987 (95% CI, 0.984-0.989) for RCT classification. We adjusted the parameters of RobotSearch to perform a sensitive search to increase the proportion of RCTs that are correctly identified.^15^ Studies classified as likely to be RCTs were sourced for the full-text systematic review.

#### Full-Text Systematic Review: Adaptive Design Methods

We used Recoll for Windows to perform a full-text systematic review on our dialysis randomized clinical trial search results from PubMed and ClinicalTrials.gov. Recoll is based on the Xapian search engine library and provides a powerful text extraction layer and a graphical interface. The adaptive design search terms were adapted from Bothwell et al,^20^ 2018, and included phase 2/3, treatment switching, biomarker adaptive, biomarker adaptive design, biomarker adjusted, adaptive hypothesis, adaptive dose finding, pick the winner, drop the loser, sample size re-estimation, re-estimations, adaptive randomization, group sequential, adaptive seamless, adaptive design, interim monitoring, Bayesian adaptive, flexible design, adaptive trial, play the winner, adaptive method, adaptive and dose and adjusting, response adaptive, adaptive allocation, adaptive signature design, treatment adaptive, covariate adaptive, and sample size adjustment (Table S2).

#### Manual Full-Text Review

**Figure 1:**
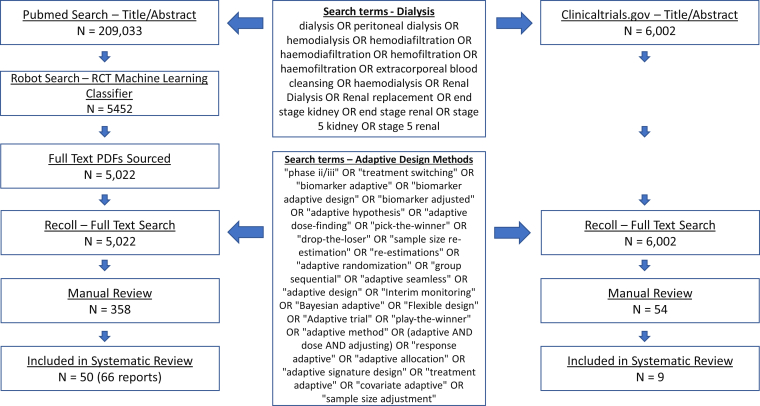
Preferred Reporting Items for Systematic Review and Meta-analysis (PRISMA) flow diagram. Abbreviation: RCT, randomized clinical trial.

We then performed manual full-text review to confirm studies that were included in the final systematic review. This process is summarized in a PRISMA flowchart (Fig 1). Full-text review was performed by C.J., R.M., and C.R. Disagreements were resolved by consensus and when a resolution was not reached by discussion, a consensus was reached through a third reviewer (M.J.O.).

### Inclusion/Exclusion Criteria for the Selection of Studies

#### Type of Study Design and Participants

RCTs of interventions in patients with kidney failure requiring dialysis and acute kidney injury (AKI) undergoing kidney replacement therapy including hemodialysis, peritoneal dialysis, hemodiafiltration, and hemofiltration. We did not limit our population to any specific disease. Additionally, we included studies that included dialysis as either a primary or secondary outcome.

#### Type of Intervention and Outcome

We did not place a restriction on the intervention type and included trials that studied medications during dialysis, medical devices, dialysis parameters, and dialysis modality. Dialysis parameter is any specification of the dialysis treatment that can be changed at each session, for example, duration, ultrafiltration rate, and sodium profiling. We included all outcomes including surrogate markers, patient-centered outcomes, and hard clinical outcomes.

#### Selection and Analysis of Trials

C.J., R.M., and C.R. extracted the study characteristics independently and in parallel. Data collected included type of the adaptive design, stopping rule, impact of adaptive design (ie, stopping for futility or efficacy and sample size changes), trial population, intervention, dialysis modality, the country of the lead investigator, and the funder of the study (adapted from Hatfield et al,^21^ 2016; Table S3).

#### Assessment of the Quality of the Studies: Risk of Bias

We used the Cochrane Risk of Bias 2 Tool^22^ to assess methodological quality of eligible trials, including random sequence generation, allocation concealment, blinding of participants and health care personnel, blinded outcome assessment, completeness of outcome data, evidence of selective reporting, and other biases. Risk-of-bias assessments were performed independently by C.J., R.M., C.R., and S.C. and disagreements were resolved by consensus. If 1 or more domains was rated as high, the study was considered at high risk of bias. We summarized our findings in a risk-of-bias table using the revised Cochrane risk-of-bias tool for randomized trials^23^ (Table S4).

### Data Synthesis

A descriptive synthesis of the data was performed. We reported overall outcomes and outcomes by: (1) frequency and type of adaptive design; (2) adaptive designs as a proportion of studies classified as dialysis RCTs by RobotSearch; (3) population, intervention, and outcome, including dialysis modality (hemodialysis, peritoneal dialysis, hemodiafiltration, and hemofiltration); (4) publication in high-impact journals; (5) geographic location and funding; (6) reporting of adaptive design methods in title and abstract; and (7) a risk-of-bias assessment.

## Results

The systematic search of articles on MEDLINE (PubMed) with dialysis keywords published before June 1, 2020, identified 209,033 results. A total of 5,452 articles were classified as probable RCTs by the machine learning classifier RobotSearch.^15^ Full-text articles were sourced (n = 5,022) and we performed a full-text systematic review using adaptive design keywords that identified 358 studies for manual screening. A total of 50 studies, available as 66 articles, were included after full-text review (Fig 1). The systematic search of ClinicalTrials.gov with dialysis keywords published before June 1, 2020, identified 6,002 registered studies. A systematic search of ClinicalTrials.gov summary files using adaptive design keywords identified 54 studies for full review and 9 studies were included. In total, 57 studies, available as 68 articles and 7 ClinicalTrials.gov summaries, were included in the final analysis. A total of 31 studies were conducted in dialysis populations and 26 studies included dialysis as a primary or secondary outcome.

### Study Characteristics

#### Frequency and Type of Adaptive Design

**Figure 2:**
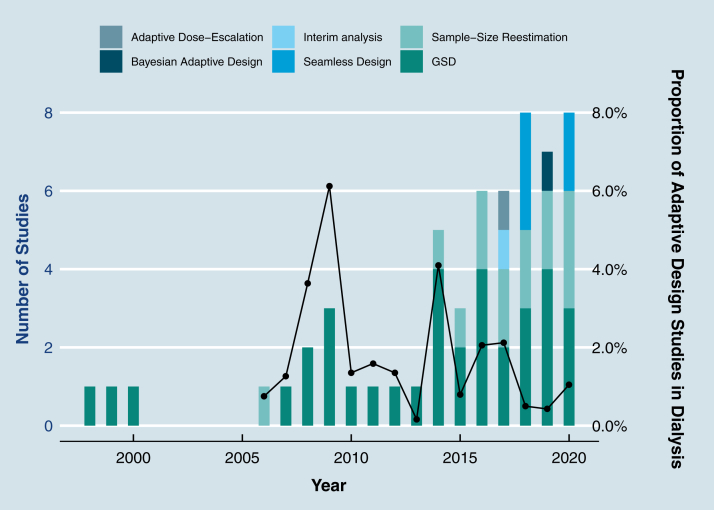
Adaptive design in dialysis randomized clinical trials by year. Abbreviation: GSD, group sequential design.

Figure 2 reports the number of adaptive designs by year and alongside the proportion of all dialysis RCTs that used adaptive design methods. The absolute amount of dialysis trials using adaptive designs has increased each year but this has not matched the overall increase in dialysis trials and resulted in a relative decrease over time in the use of adaptive design methods in dialysis trials, ranging from 6.12% in 2009 to 0.43% in 2019, with a mean of 1.82%. A 1-way analysis of var1ance was conducted to determine whether the proportion of adaptive trials was different by year. Adaptive trials proportion was statistically significantly different between years, *F*_17_ = 3.391; *P* < 0.001. Tukey post hoc analysis revealed statistically significant differences between 2009 and 2013 (−5.96 [95% CI, −10.73 to −1.19]; *P* = 0.002); 2019 (−5.7 [95% CI, −10.36 to −1.04]; *P* = 0.003); 2018 (−5.62 [95% CI, −10.29 to −0.96]; *P* = 0.003), 2015 (−5.33 [95% CI, −10.21 to −0.45]; *P* = 0.02), 2020 (−5.07 [95% CI, −9.81 to −0.34]; *P* = 0.021]; and between 2014 and 2019 (−3.67 [95% CI, −6.69 to −0.65]; *P* = 0.003) and 2018 (−3.6 [95% CI, −6.62 to −0.58]; *P* = 0.004).

Group sequential designs were the most common type of adaptive design method used; 35 (61.4%) trials (22 [71%] in dialysis populations and 13 [50%] in dialysis outcome trials; Table 1^24^, ^25^, ^26^, ^27^, ^28^, ^29^, ^30^, ^31^, ^32^, ^33^, ^34^, ^35^, ^36^, ^37^, ^38^, ^39^, ^40^, ^41^, ^42^, ^43^, ^44^, ^45^, ^46^, ^47^, ^48^, ^49^, ^50^, ^51^, ^52^, ^53^, ^54^, ^55^, ^56^, ^57^, ^58^, ^59^, ^60^, ^61^, ^62^, ^63^, ^64^, ^65^). The O’Brien-Fleming stopping boundary was the most common stopping rule, used in 9 trials (25.7%), followed by Lan DeMets, used in 8 trials (22.9%). A total of 29 trials (50.9%) were affected by the use of group sequential adaptive design, including 7 trials (41.2%) that stopped early for futility, 3 trials (17.6%) that stopped early for efficacy, and 4 trials (23.5%) that stopped early for safety.

**Table 1:** Group Sequential Trials in Dialysis Randomized Clinical Trials

Study	Stopping Rule	Impact of Adaptive Design	Population	Intervention	Primary Outcome	Nature of Primary Outcome	Dialysis Modality	Sample Size of Study	Country	Funder Type	Funder	Study Phase
**AKI**												
Acker et al^24^ (2000)	Pocock	Significant difference in mortality observed at first analysis; trial terminated	Patients with acute kidney failure	Thyroxine	Medication	Percentage requiring dialysis	HD/HF	59	US	NR	NR	Phase 3
ATN^25^^,^^96^ (2008)	Haybittle-Peto rule	2 interim analyses performed as planned, trial continued per protocol	Critically ill patients with AKI and failure of at least 1 nonrenal organ or sepsis	Intensive or less intensive KRT	Dialysis parameter	Death from any cause by d 60	HD/HF	1,124	US	Public	Cooperative studies program VA & NIDDK	Phase 3
Ejaz et al^26^ (2009)	Z boundary	Study stopped after completion of stage	Patients undergoing high-risk cardiac surgery	Nesiritide	Medication	Dialysis and/or all-cause mortality within 21 d	HD	94	US	Private	Scios Inc	Phase 3
IVOIRE^27^ (2013)	NR	1 interim analysis performed as planned, trial d/c due to difficulty recruiting	Critically ill patients with septic shock and AKI	HVHF	Dialysis modality	28-d mortality	HF	140	France	Public	French Health Ministry	Phase 3
FENO HSR^28^ (2014)	Reboussin et al and Lan DeMets stopping rule	Stopped due to futility after interim analysis 3	Critically ill cardiac surgery patients with AKI	Fenoldopam	Medication	Rate of KRT	Any KRT	667	Italy	Public	Italian Ministry of Health	Phase 3
FBI^29^ (2014)	Fleming-Harrington (O Brien-Fleming boundary)	Trial not complete	Critically ill patients with AKI receiving CKRT	Enoxaparin	Medication	Occurrence of venous thromboembolism	HD/HDF/HF	266	Denmark	Public	Danish society of anesthesiology; intensive medicines research initiative	Phase 3
HEROICS^30^ (2015)	Triangular test (Whitehead 1978)	At sequential interim analysis 3 trial was stopped for futility	Patients with severe shock requiring high-dose catecholamines 3-24 h post–cardiac surgery	Early HVHF	Dialysis modality	30-d mortality	HF/HDF	224	France	Public and private	French Ministry of Health; Hospal-Gambro	Phase 3
AKIKI^31^^,^^32^ (2016)	O Brien-Fleming boundary	2 interim analyses before final analysis; no change to trial	Patients with severe AKI requiring mechanical ventilation, catecholamine infusion, or both	Early or delayed strategy of KRT	Dialysis parameter	Overall survival at d 60	HD	620	France	Public	French Ministry of Health	Phase 3
ELAIN Trial^33^^,^^34^ (2016)	O Brien-Fleming boundary	1 interim analysis performed after half of total no. of deaths across both treatment groups; no change to trial	Critically ill patients with AKI and plasma NGAL level > 150 ng/mL	Early or delayed initiation of KRT	Dialysis parameter	Mortality at 90 d	HD/HDF/HF	231	Germany	Private	Else-Kroner Fresenius Stiftung	Phase 3
LEVO-CTS^35^^,^^97^ (2017)	O Brien-Fleming boundary	NR	Patients with EF < 35% undergoing cardiac surgery with cardiopulmonary bypass	IV levosimendan	Medication	Composite of 30-d mortality, KRT, perioperative MI, or mechanical cardiac assist device through d 5	HD/HDF	882	US	Private	Tenax Therapeutics	Phase 3
CULPRIT-SHOCK^36^^,^^37^ (2018)	O Brien-Fleming boundary	NR	Patients with cardiogenic shock complicating acute MI	Culprit lesion only, primary coronary intervention	Treatment strategy	30-d mortality or AKI requiring KRT	HD/HDF	706	Germany	Public	EU; German Heart Research Foundation; German Cardiac Society	Phase 3
PRESERVE^38^ (2018)	O Brien-Fleming boundary	Sponsor stopped trial after prespecified interim analysis due to absence of between-group difference	Patients at high risk for kidney complications scheduled for angiography	1.26% sodium bicarbonate or IV 0.9% sodium chloride and 5 d of oral acetylcysteine or oral placebo	Medication	Composite of death, need for dialysis, or persistent increase of at least 50% from baseline in Scr at 90 d	HD	5,177	US	Public	US Dept of VA Office of Research and Development; National Health and Medical Research Council of Australia	Phase 3
VIOLET^39^ (2018)	Lan DeMets	Study stopped for futility after interim analysis 1	Acute respiratory distress syndrome, vitamin D deficiency, and critical illness	Vitamin D_3_	Medication	90-d all-cause mortality	HD	1,358	US	Public	NHLBI	Phase 3
Schanz et al^40^ (2019)	Jennison and Turnbull	Study stopped prematurely after interim analysis due to futility	Patients at high risk for AKI	Screened with urinary [TIMP-2][IGFBP7]	Other	Incidence of moderate to severe AKI within the first d after admission	HD	100	Germany	Public	Robert-Bosch-Foundation	Phase 3
HYVITS (NCT03380507) (2019)	O Brien-Fleming boundary	Trial not complete	Septic shock and critical illness	Hydrocortisone, vitamin C, and thiamine	Medication	Hospital mortality at 60 d	HD	212	Qatar	Industry	Hamad Medical Corp	Phase 2/3
RICH^41^^,^^42^ (2020)	O Brien-Fleming boundary	Stopped early for efficacy	Critically ill patients with AKI	Regional citrate anticoagulation compared with systemic heparin anticoagulation	Dialysis parameter	Filter life span and 90-d mortality	HDF	596	Germany	Public	German Research Foundation	Phase 3
REMOVE (NCT03266302) (2020)	Pocock	Trial not complete	Infective endocarditis	Hemoadsorber for removal of cytokines	Medical device	Change in mean total SOFA score	HD	288	Germany	Public and private	German	
Federal Ministry of Education and Research; CytoSorbents Europe GmbH	Phase 2											
**Kidney Failure Requiring Dialysis**												
Besarab et al^43^ (1998)	Lan-DeMets	Trial stopped at interim analysis 3 due to concerns about safety	HD patients with clinical evidence of congestive heart failure or ischemic heart disease	Epoetin and target hematocrit	Medication	Time to death or first nonfatal MI	HD	1,233	US	Private	Amgen	Phase 3
ACTION II^44^ (1999)	Lan-DeMets	Terminated enrollment due to unfavorable perceived risk-benefit ratio	T2DM patients with kidney disease	Aminoguanidine	Medication	Doubling of Scr concentration	HD	900	US	NR	NR	Phase 3
Chapman et al^45^ (2007)	Constrained stopping boundaries	2 interim analyses, trial continued	Liver resection, spine, peripheral arterial bypass, and dialysis access surgery	Recombinant human thrombin (rhThrombin)	Medication	Time to hemostasis	HD	76	US	Private	ZymoGenetics, Inc	Phase 3
DAC^46^ (2008)	Lan DeMets	Enrollment stopped after 877 patients randomized based on stopping rule for intervention efficacy	Participants with ESKD undergoing new fistula creation	Clopidogrel	Medication	Fistula thrombosis	HD	877	US	Public	NIDDK; NIH	Phase 3
DAC^47^ (2009)	Lan DeMets	5 planned interim analyses performed before final analysis; no change to trial	Participants with placement of a new arteriovenous graft	Extended-release dipyridamole plus aspirin	Medication	Loss of primary unassisted patency	HD	649	US	Public and private	NIDDK; NIH; Boehringer Ingelheim	Phase 3
AURORA^48^^,^^49^ (2009)	Event driven	Continuation of study was recommended by data and safety monitoring board	Maintenance HD patients	Rosuvastatin	Medication	Death from cardiovascular causes, nonfatal MI, or nonfatal stroke	HD	2,776	Sweden	Private	AstraZeneca	Phase 3
ACCORD^50^ (2010)	Lan DeMets	Intensive therapy stopped before study end due to increased mortality	Volunteers with established T2DM, HbA_1c_ ≥ 7.5%, and CVD or ≥2 CVD risk factors	Target HbA_1c_ < 6.0%.	Treatment target	Dialysis or kidney transplantation or Scr > 291.7 μ/L or retinal photocoagulation or vitrectomy	HD	10,251	US	Public	NHLBI	Phase 3
OPPORTUNITY^51^^,^^52^ (2011)	Event-driven	Trial terminated early due to slow recruitment	Adult maintenance HD patients	Recombinant human growth hormone	Medication	Mortality	HD	695	US	Private	Novo Nordisk	Phase 3
CONTRAST^53^^,^^54^ (2012)	Double triangular test (Whitehead 2007)	Board recommended to stop trial as enough evidence was provided for futility	Patients with ESKD	Online HDF	Dialysis modality	All-cause mortality	HD/HDF	714	the Netherlands	Public and private	Dutch Kidney Foundation; Fresenius Medical Care; Gambro Lundia	Phase 3
HONEYPOT^55^^,^^56^ (2014)	Haybittle-Peto rule	Stopping rule for efficacy not met and study was completed as per protocol	PD patients	Daily topical exit-site application of antibacterial honey	Medication	Time to first infection related to PD	PD	371	Australia	Public and private	Baxter Healthcare; Queensland Government; Comvita; Gambro	Phase 3
HALT-PKD^57^ (2014)	Lan DeMets	Study extended due to lower-than-expected no. of end points	Patients with ADPKD	Lisinopril and telmisartan	Medication	Time to death, ESKD, or 50% reduction from baseline eGFR.	HD	486	US	Public	NIDDK	Phase 3
Knoll et al^58^^,^^59^ (2015)	O Brien-Fleming boundary	Extended follow-up to 4 y to increase statistical power due to slower-than-expected recruitment	Kidney transplant patients with proteinuria and eGFR of 20-55 mL/min/1.73 m^2^	Ramipril	Medication	Doubling of Scr, ESKD, or death	HD	528	Canada	Public	Canadian Institutes of Health Research	Phase 3
PAVE^60^ (2016)	Lan DeMets	Trial not complete	Patients with native arteriovenous fistula	Paclitaxel-coated balloons	Medical device	Time to end of target lesion primary patency	HD	211	UK	Public	National Institute for Health Research EME programme	Phase 3
OPN-305 (NCT01794663) (2016)	NR	Unknown	Kidney transplant recipients with delayed graft function	OPN-305 (tomaralimab)	Medication	Measure of early graft function	HD	252	Ireland	Industry	Opsona Therapeutics Ltd	Phase 2
FAVOURED^61^^,^^62^ (2017)	Haybittle-Peto rule	Early cessation of recruitment, only interim analysis 1 was performed	Participants with stage 4 or 5 CKD after arteriovenous fistula creation	Fish oil supplementation	Medication	Fistula failure, a composite of fistula thrombosis and/or abandonment and/or cannulation failure, at 12 mo	HD	567	Australia	Public and private	National Health and Medical Research Council of Australia; Amgen Australia Pty Ltd; Mylan EPD	Phase 3
CREDENCE^63^ 2019)	Alpha spending function	Prespecified efficacy criteria for early cessation were achieved so board recommended that trial be stopped	Patients with T2DM and albuminuric CKD	Canagliflozin	Medication	Composite of ESKD (dialysis, transplantation, sustained GFR < 15), doubling of Scr, or death from kidney or cardiovascular causes	HD	4,401	Australia	Private	Janssen Research and Development	Phase 3
DECLARE-TIMI 58^64^ (2019)	O Brien-Fleming boundary	2 interim analyses performed; no change to trial	Patients with T2DM who had or were at risk for atherosclerotic CVD	Dapagliflozin	Medication	Cardiovascular death, MI, or ischemic stroke or hospitalization for heart failure	HD	17,160	US	Private	AstraZeneca	Phase 3
CONVINCE^65^ (2020)	Haybittle-Peto rule	Trial not complete	Patents with ESKD treated with HD	High-dose HF conventional high-flux HD	Dialysis modality	All-cause mortality	HD/HDF	1,800	the Netherlands	Public	European Union's Horizon 2020 research and innovation programme	Phase 3

Abbreviations: ADPKD, autosomal dominant polycystic kidney disease; AKI, acute kidney injury; CKD, chronic kidney disease; CKRT, continuous kidney replacement therapy; CVD, cardiovascular disease;; d/c, discontinued; EF, ejection fraction; eGFR, estimated glomerular filtration rate; ESKD, end-stage kidney disease; HbA1c, glycated hemoglobin; HD, hemodialysis; HDF, hemodiafiltration; HF, hemofiltration; HVHF, high-volume hemofiltration; IGFBP7, insulin like growth factor binding protein 7; IV, intravenous; KRT, kidney replacement therapy; MI, myocardial infarction; NGAL, neutrophil gelatinase-associated lipocalin; NHLBI, National Heart, Lung, and Blood Institute; NIDDK, National Institute of Diabetes and Digestive and Kidney Diseases; NIH, National Institutes of Health; NR, not reported; PD, peritoneal dialysis; Scr, serum creatinine; SOFA, Sequential Organ Failure Assessment; T2DM, type 2 diabetes mellitus; VA, Veterans Administration.

Sample-size re-estimation was the second most common type of adaptive design, used in 14 trials (24.6%); 8 (25.8%) in dialysis populations and 6 (23.1%) in dialysis outcome trials (Table 2^66^, ^67^, ^68^, ^69^, ^70^, ^71^, ^72^, ^73^, ^74^, ^75^, ^76^, ^77^, ^78^, ^79^, ^80^, ^81^, ^82^). Eight trials (57.1%) were affected by the use of sample-size re-estimation adaptive design including 6 trials (75%) that increased sample size.

**Table 2:** Sample-Size Re-estimation in Dialysis Randomized Clinical Trials

Study	Impact of Adaptive Design	Population	Intervention	Primary Outcome	Nature of Primary Outcome	Dialysis Modality	Sample Size	Country	Funder Type	Funder	Study Phase
**AKI**											
Hemodiafe^66^ (2006)	Sample size adjusted to include 180 patients per group	Critically ill patients with acute kidney failure as part of multiple-organ dysfunction syndrome	Intermittent HD vs CVHDF	Dialysis modality	60-d survival	HD/HDF	360	France	Public	Societe de Reanimation de Langue Francaise	Phase 4
Riley et al^67^ (2014)	Data from initial 10 randomized patients demonstrated >50% difference in urine output, revealing adequate power would be achieved with only 20 randomized patients	Infants < 90 d old with congenital heart disease who underwent bypass surgery and were postoperatively treated with CPD	Continue 24 h more CPD or discontinue CPD	Dialysis modality	Urine output (mL/kg per h)	PD	20	US	Public	Baylor College of Medicine; Cincinnati Children - Hospital Medical Center	Phase 3
SCD^68^ (2015)	Study terminated by sponsor at interim analysis because SCD treatment was often outside the recommended iCa range and therefore resulted in ineffective therapy	ICU patients with AKI	Selective cytopheretic device	Medical device	60-d mortality	HDF	134	US	Private	CytoPherx, Inc.	Phase 3
TARTARE-2S^69^ (2016)	Trial not complete	Patients with septic shock	Targeted tissue perfusion vs macrocirculation-guided standard care	Treatment strategy	Alive at 30 d with normal arterial blood lactate and without inotropic or vasopressor agent	HD/HDF/HF	200	Switzerland	Public	Sigrid Juselius Foundation; Instrumentarium Foundation; Helsinki University Hospital	Phase 3
Kwiatkowski et al^70^ (2017)	NR	Infants after congenital heart surgery	PD	Dialysis modality	Negative fluid balance	PD	73	US	Public	American Heart Association Great Rivers Affiliate; internal funding from Cincinnati Children’s Hospital Medical Center	Phase 2
ANDROMEDA-SHOCK^71^ (2018)	Trial not complete	Patients with septic shock	Peripheral perfusion-targeted resuscitation	Other	28-d mortality	HD/HDF/HF	422	Chile	Public	Departamento de Medicina Intensiva, Pontificia Universidad Catolica de Chile	Phase 3
COACT^72^^,^^73^ (2019)	After interim analysis, data and safety monitoring committee advised that sample size not be increased	Post–cardiac arrest patients without signs of STEMI	Immediate coronary angiography and percutaneous coronary intervention	Treatment strategy	90-d mortality	HD/HDF	552	the Netherlands	Public	Netherlands Heart Institute	Phase 3
FRESH^74^ (2020)	Continue enrollment to increase sample size to maximum of 210 patients	Patients presenting to the ED with sepsis or septic shock and anticipated ICU admission	Dynamic assessment of fluid responsiveness (passive leg raise)	Treatment strategy	Difference in positive fluid balance at 72 h or ICU discharge	HD/HDF/HF	124	US	Private	Cheetah Medical	Phase 3
**CKD**											
PREDICT^75^^,^^76^ (2020)	Sample size amended from 220 to 238 for each group	Patients with CKD without diabetes	High and low hemoglobin groups (darbepoetin alfa)	Medication	Kidney composite end point (starting maintenance dialysis, kidney transplantation, eGFR < 6 mL/min/1.73 m^2^, and 50% reduction in eGFR)	HD	491	Japan	Private	Kyowa Hakko Kirin; Otsuka; Dainippon Sumitomo; Mochida	Phase 3
**Kidney Failure Requiring Dialysis**											
Kratochwill et al^77^ (2016)	Led to premature termination of patient recruitment	Stable PD outpatients	Alanyl-glutamine addition to glucose-based PD fluid	Medication	Heat-shock protein 72 expression	PD	20	Austria	Public	ZIT - Technology Agency of the City of Vienna; FFG - the Austrian Research Promotion Agency	Phase 2
IDPN-Trial^78^ (2017)	Sample size was increased; primary outcome was significant	Maintenance HD patients with protein-energy wasting	IDPN	Medication	Prealbumin	HD	107	Germany	Private	Fresenius Kabi Germany GmbH	Phase 4
CHART^79^^,^^80^ (2018)	Sample-size re-estimation not performed	Urologic patients undergoing elective cystectomy	Albumin 5% or balanced hydroxyethyl starch 6%	Medication	Ratio of serum cystatin C between last visit at d 90 and t preoperative visit 1	HD	100	Germany	Private	CSL Behring GmbH	Phase 3
KALM-1^81^ (2019)	NR	HD patients with moderate to severe pruritus	Intravenous difelikefalin	Medication	24-h Worst Itching Intensity Numerical Rating Scale	HD	378	US	Private	Cara Therapeutics	Phase 3
Fujimoto et al^82^ (2020)	Sample size calculated by intermediate analysis of first 30 samples enrolled	Patients on maintenance HD 3×/wk	Lidocaine/prilocaine cream (EMLA)	Medication	Puncture pain relief, measured using a 100-mm visual analog scale	HD	66	Taiwan	Public	Grant-in-aid for Young Scientists from the Japan Society for the Promotion of Science	Phase 2

Abbreviations: AKI, acute kidney injury; CKD, chronic kidney disease; CPD, continuous peritoneal dialysis; CVHDF, continuous venovenous hemodiafiltration; ED, emergency department; eGFR, estimated glomerular filtration rate; HD, hemodialysis; HDF, hemodiafiltration; HF, hemofiltration; iCa, ionized calcium; ICU, intensive care unit; IDPN, intradialytic parenteral nutrition; NR, not reported; PD, peritoneal dialysis; SCD, selective cytopheretic device; STEMI, ST-elevation myocardial infarction.

Phase 2/3 seamless design was the third most common type of adaptive design; 5 trials (8.8%); 1 (3.23%) in dialysis populations and 4 (15.4%) in dialysis outcome trials (Table 3^83^, ^84^, ^85^, ^86^, ^87^, ^88^, ^89^). Adaptive dose-escalation, Bayesian adaptive design, and interim analysis were used in 1 trial each.

**Table 3:** Seamless Design/Adaptive Dose Escalation in Dialysis Randomized Clinical Trials

Study	Impact of Adaptive Design	Population	Intervention	Primary Outcome	Nature of Primary Outcome	Dialysis Modality	Sample Size	Country	Funder Type	Funder	Study Phase
**Phase 2a/2b Seamless Design**											
STOP-AKI^83^^,^^84^ (2018)	Combined efficacy and dose-finding study	Critically ill patients with sepsis-associated AKI	Human recombinant alkaline phosphatase	Medication	Area under the time-corrected endogenous creatinine clearance curve from d 1-7	HD	301	the Netherlands	Private	AM-Pharma	Phase 2a/2b
**2-Stage Seamless Adaptive Design**											
Himmelfarb et al^85^ (2018)	At end of each stage, data from patients are used to select the THR-184 dose arms for next stage	Patients at high risk for AKI after cardiac surgery	THR-184	Medication	Proportion of patients who developed AKI	HD/HDF/HF	452	US	Private	Thrasos Therapeutics, Inc	Phase 2
**Adaptive Phase 2b/3**											
SEPSIS-ACT^86^ (2018)	Trial was stopped for futility at end of part 1	Septic shock requiring >5 μg/min of norepinephrine	Selepressin	Medication	Vasopressor- and mechanical ventilator-free days (PVFDson)	HD	868	US	Industry	Ferring Pharmaceuticals	Phase 2/3
**Phase 2/3 Seamless Design**											
COMBAT-SHINE^87^ (2020)	Trial not complete	Patients with septic shock–induced endotheliopathy	Infusion of iloprost	Medication	Mean daily modified Sequential Organ Failure Assessment score	HD	384	Denmark	Public	Danish Independant Research Organisation	Phase 2
Cohen et al (NCT04381052) (2020)	Trial not complete	Patients with life-threatening COVID-19	Clazakizumab	Medication	Cumulative incidence of serious adverse events associated with clazakizumab or placebo	Any	30	US	Public and private	Columbia University; NYU Langone Health; CSL Behring	Phase 2
**Adaptive Dose-Escalation**											
EMPIRIKAL^88^ (2017)	Trial not complete	Patients after receiving deceased donor kidney transplants	Mirococept	Medication	Delayed graft function	HD/HDF/HF	560	UK	Public	Medical Research Council	Phase 2
**Bayesian Adaptive Design**											
ASTOUND (NCT02723591) (2019)	Trial shortened to 1 y due to a stopping rule	Kidney transplantation	Tacrolimus	Medication	Percentage of participants positive for de novo DSA or immune activation occurrence	HD	599	US	Industry	Astellas Pharma Inc	Phase 4
**Interim Analysis**											
Hosgood et al^89^ (2017)	Trial not complete	Patients receiving kidney from donation after circulatory death donor	Ex vivo normothermic perfusion	Other	Rates of delayed graft function defined as need for dialysis in first wk posttransplantation	HD	400	UK	Public	Kidney Research UK; University of Cambridge and University Hospitals of Cambridge Foundation Trust.	Phase 2

Abbreviations: AKI, acute kidney injury; COVID-19, coronavirus disease 2019; DSA, donor-specific antibody; HD, hemodialysis; HDF, hemodiafiltration; HF, hemofiltration; PD, peritoneal dialysis.

#### Population, Intervention, and Outcome Studied

**Figure 3:**
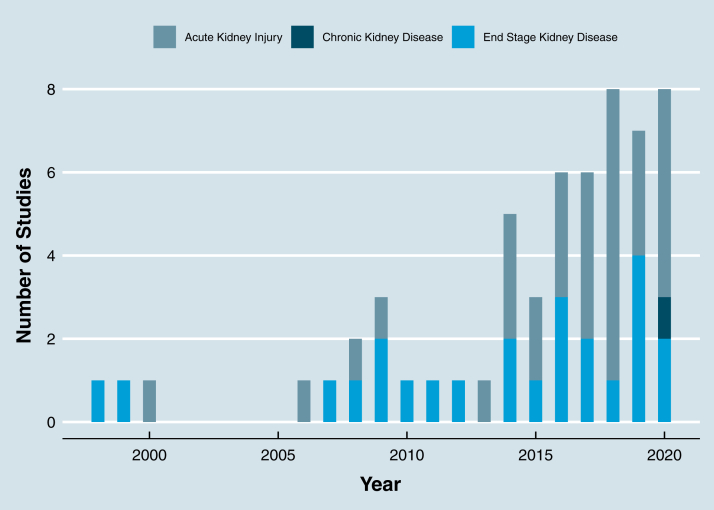
Populations with adaptive design in dialysis randomized clinical trials by year.

AKI was studied in 32 trials (56.1%), kidney failure requiring dialysis was studied in 24 trials (42.1%), and chronic kidney disease (CKD) was studied in 1 trial (1.75%). Figure 3 reports the number of each population under study per year and shows a larger increase in adaptive design methods in AKI populations compared with kidney failure requiring dialysis populations. Medications were the most common intervention type, evaluated in 35 trials (61.4%), followed by dialysis modality in 7 trials (12.3%) and dialysis parameter in 4 trials (7%). Hemodialysis was the most common dialysis modality studied in 32 trials (56.1%), followed by hemodialysis and hemodiafiltration in 8 trials (14%); hemodialysis, hemodiafiltration, and hemofiltration in 7 trials (12.3%); and peritoneal dialysis in 4 trials (7%). Hard clinical outcomes were selected in 34 trials (59.6%), followed by surrogate outcomes in 20 trials (35.1%) and mixed in 3 trials (5.3%). The outcome measure was continuous in 15 trials (26.3%) and dichotomous in 42 trials (73.7%). Phase 3 studies were the most common study phase, studied in 41 trials (71.9%; Table 1, Table 2, Table 3).

#### Publication in High-Impact Journals

A total of 32 studies (56.1%) were published in a high-impact journal (impact factor > 9). Fourteen studies (24.6%) were published in the *New England Journal of Medicine*, 6 studies (10.5%) were published in the *Journal of the American Medical Association*, 4 studies (7%) were published in *Trials*, and 2 studies (3.5%) were published in the *Journal of the American Society of Nephrology*.

#### Geographic Location and Funding

The most common country of the lead author was the United States in 24 studies (42.1%), followed by Germany in 7 studies (12.3%), France in 4 studies (7%), the Netherlands in 4 studies (7%), Australia in 3 studies (5.3%), and the United Kingdom in 3 studies (6%; Table 1, Table 2, Table 3). Forty-nine studies (86%) were multicenter trials. Twenty-seven studies (47.4%) were supported by public funding, 21 studies (36.8%) were supported by private funding, 7 studies (12.3%) were supported by both public and private funding, and 2 studies (3.5%) did not report the source of funding.

### Reporting of Adaptive Design Method in Title and Abstract

A total of 44 studies (77.2%) did not report their adaptive design method in the title or abstract and would not be detected by a standard systematic review search.

### Risk of Bias

Risk of bias was assessed for 40 trials (protocols and clinicaltrials.gov were excluded; Fig S1; Table S4). Overall risk of bias was deemed to be “low” in 17 trials (42.5%), “some concerns” in 13 trials (32.5%), and “high risk” in 10 trials (25%). The randomization process led to some concerns for 10 studies (25%). Deviations from intended interventions led to some concerns for 4 studies (10%) and high risk for 6 studies (15%). Missing outcome data were deemed to be some concerns for 2 studies (5%) trials and high risk of bias for 2 studies (5%). Measurement of outcome measures was deemed to be some concerns for 2 studies (5%) trials and high risk of bias for 1 study (2.5%). Selection of the reported result was deemed to be some concerns for 6 studies (15%) trials and high risk of bias for 1 study (2.5%).

## Discussion

In this systematic review, we report that adaptive design methods were used in 57 dialysis RCTs over a 20-year period. Although the absolute number has increased over time, the relative use of adaptive design methods in trials in dialysis populations and trials with dialysis as an end point has decreased.

First, we report that the relative proportion of adaptive design methods in dialysis trials has decreased over time. The absolute number of dialysis trials using adaptive designs has increased each year, but this has not matched the overall increase in dialysis trials and therefore resulted in a relative decrease. We were unable to compare this result with other specialties because recent systematic reviews have not reported the relative use of adaptive designs.^21^^,^^90^

Second, we report that group sequential designs are the most used type of adaptive design in dialysis trials. This is similar to previous systematic reviews in cardiology^91^ and oncology^90^ and in a review of registered clinical trials covering multiple specialties on clinicaltrials.gov.^21^

Third, we report that adaptive designs were more common in AKI (56.1% of trials) than kidney failure requiring dialysis (42.1% of trials). This may reflect increasing use of adaptive design methodology in critical care^92^ and sepsis-related trials,^93^ in which AKI is most common. There were very few trials of CKD with a dialysis outcome (2%) that used an adaptive design. Many reasons for the paucity of CKD trials have been previously suggested, including the use of treatments in CKD despite a lack of evidence, difficulty recruiting to CKD trials due to stringent eligibility criteria, and underpowered subgroup analysis.^4^^,^^94^ The infrequent use of adaptive designs in CKD trials may become a self-perpetuating barrier to using adaptive designs in future trials.^21^

Fourth, we report that adaptive design methods affected the conduct of the randomized trial in most studies (50.9%). For example, 17 (48.6%) trials were affected by the use of group sequential adaptive design, including 7 trials (41.2%) stopped early for futility, 3 trials (17.6%) stopped early for efficacy, and 4 trials (23.5%) stopped early for safety. This finding is similar to a systematic review of published and publicly available trials in which the most common reason for stopping group sequential trials was futility.^20^

Fifth, we found that the most common country of the lead author was the United States, 24 studies (42.1%), and the most common funding source was public, 27 studies (47.4%). This finding was different from a systematic review of published and publicly available trials in which 65% of trials reported industry funding.^20^ Funding for kidney research reached an all-time low in 2013^5^ but this has recently changed in the United States with advocacy from scientific societies such as the American Society of Nephrology, whereby an executive order was signed in 2020 to reform the US end-stage kidney disease treatment industry.^95^ Adaptive designs are one part of the solution for optimizing the design of clinical trials in dialysis and nephrology and will benefit from the improvement in the funding landscape.^94^

Our study has several limitations. First, we limited our search to 2 databases (PubMed and ClinicalTrials.gov) due to the scale of studies sourced (209,033 and 6,002 results). This was a deviation from our protocol but necessary to make this full-text review feasible. Second, we decided to include RCTs with dialysis outcomes in addition to patients currently receiving dialysis. This permitted a more comprehensive review of the full landscape of AKI, kidney failure requiring dialysis, and CKD trials, but was a deviation from our original protocol. Third, the denominator for calculating the proportion of adaptive designs in all dialysis RCTs will include some false positives, that is, either not RCTs or not dialysis. We modified the parameters of the machine learning classifier to perform a sensitive search to include as many true positives as possible. We expect this misclassification bias to be independent of time and bias every year equally and therefore not affect the trend. Fourth, publication bias, in which negative studies are not published, will bias out results toward the null, for example, our estimate of the impact of adaptive design (50.9%) would be higher if unpublished studies stopped for futility and not published were included.

In summary, we developed a novel full-text systematic review search strategy. Forty-four studies (77.2%) did not report their adaptive design method in the title or abstract and would not be detected by a standard systematic review search methodology. This could introduce a reporting bias in which adaptive design methods are reported in the main article but not in the abstract. Our novel strategy combined classical systematic review, machine learning classifiers, and a novel full-text systematic review. This new method has broad applications in medical evidence synthesis and evidence synthesis in general.

Adaptive design methods improve the efficiency of RCTs in dialysis but their relative use in dialysis is decreasing over time. Greater knowledge of adaptive design examples in dialysis will further improve uptake in dialysis RCTs.

## Supplementary Material

Supplementary File (PDF) Figure S1, Item S1, Tables S1-S4.

## Footnotes

∗ This article is available as a preprint.^1^
