# Supplementary material for: Trends in Adaptive Design Methods in Dialysis Clinical Trials: A Systematic Review
Source: Kidney Med. 2021 Aug 20;3(6):925–41. doi: 10.1016/j.xkme.2021.08.001 (PMC8664746; doi:10.1016/j.xkme.2021.08.001)
Supplement: Supplementary File (PDF) — Figure S1, Item S1, Tables S1-S4. [file mmc1.pdf]

**Item S1.** Prisma Checklist

| Section/topic | # | Checklist item | Reported on page # |
|---|---|---|---|
| **TITLE** | | | |
| Title | 1 | Identify the report as a systematic review, meta-analysis, or both. | 1 |
| **ABSTRACT** | | | |
| Structured summary | 2 | Provide a structured summary including, as applicable: background; objectives; data sources; study eligibility criteria, participants, and interventions; study appraisal and synthesis methods; results; limitations; conclusions and implications of key findings; systematic review registration number. | 4 |
| **INTRODUCTION** | | | |
| Rationale | 3 | Describe the rationale for the review in the context of what is already known. | 6/7 |
| Objectives | 4 | Provide an explicit statement of questions being addressed with reference to participants, interventions, comparisons, outcomes, and study design (PICOS). | 7 |
| **METHODS** | | | |
| Protocol and registration | 5 | Indicate if a review protocol exists, if and where it can be accessed (e.g., Web address), and, if available, provide registration information including registration number. | 8 |
| Eligibility criteria | 6 | Specify study characteristics (e.g., PICOS, length of follow-up) and report characteristics (e.g., years considered, language, publication status) used as criteria for eligibility, giving rationale. | 9/10 |
| Information sources | 7 | Describe all information sources (e.g., databases with dates of coverage, contact with study authors to identify additional studies) in the search and date last searched. | 8/9 |
| Search | 8 | Present full electronic search strategy for at least one database, including any limits used, such that it could be repeated. | Supplementary appendix pg.1 |
| Study selection | 9 | State the process for selecting studies (i.e., screening, eligibility, included in systematic review, and, if applicable, included in the meta-analysis). | 10 |
| Data collection process | 10 | Describe method of data extraction from reports (e.g., piloted forms, independently, in duplicate) and any processes for obtaining and confirming data from investigators. | 10 |
| Data items | 11 | List and define all variables for which data were sought (e.g., PICOS, funding sources) and any assumptions and simplifications made. | 10/11 |
| Risk of bias in individual studies | 12 | Describe methods used for assessing risk of bias of individual studies (including specification of whether this was done at the study or outcome level), and how this information is to be used in any data synthesis. | 10 |
| Summary measures | 13 | State the principal summary measures (e.g., risk ratio, difference in means). | 10/11 |

| | | | |
|---|---|---|---|
| Synthesis of results | 14 | Describe the methods of handling data and combining results of studies, if done, including measures of consistency (e.g., $I^2$) for each meta-analysis. | N/A |
| Risk of bias across studies | 15 | Specify any assessment of risk of bias that may affect the cumulative evidence (e.g., publication bias, selective reporting within studies). | 14 |
| Additional analyses | 16 | Describe methods of additional analyses (e.g., sensitivity or subgroup analyses, meta-regression), if done, indicating which were pre-specified. | N/A |

| | | | |
|---|---|---|---|
| **RESULTS** | | | |
| Study selection | 17 | Give numbers of studies screened, assessed for eligibility, and included in the review, with reasons for exclusions at each stage, ideally with a flow diagram. | 12 |
| Study characteristics | 18 | For each study, present characteristics for which data were extracted (e.g., study size, PICOS, follow-up period) and provide the citations. | 26-30 |
| Risk of bias within studies | 19 | Present data on risk of bias of each study and, if available, any outcome level assessment (see item 12). | Supplementary appendix pg.4-6 |
| Results of individual studies | 20 | For all outcomes considered (benefits or harms), present, for each study: (a) simple summary data for each intervention group (b) effect estimates and confidence intervals, ideally with a forest plot. | N/a |
| Synthesis of results | 21 | Present results of each meta-analysis done, including confidence intervals and measures of consistency. | N/a |
| Risk of bias across studies | 22 | Present results of any assessment of risk of bias across studies (see Item 15). | Supplementary appendix pg.4-6 |
| Additional analysis | 23 | Give results of additional analyses, if done (e.g., sensitivity or subgroup analyses, meta-regression [see Item 16]). | N/a |
| **DISCUSSION** | | | |
| Summary of evidence | 24 | Summarize the main findings including the strength of evidence for each main outcome; consider their relevance to key groups (e.g., healthcare providers, users, and policy makers). | 1 |
| Limitations | 25 | Discuss limitations at study and outcome level (e.g., risk of bias), and at review-level (e.g., incomplete retrieval of identified research, reporting bias). | 1 |
| Conclusions | 26 | Provide a general interpretation of the results in the context of other evidence, and implications for future research. | 1 |
| **FUNDING** | | | |
| Funding | 27 | Describe sources of funding for the systematic review and other support (e.g., supply of data); role of funders for the systematic review. | 17 |

**Figure S1**. Risk of Bias Assessment of Dialysis Randomized Clinical Trials with Adaptive Designs

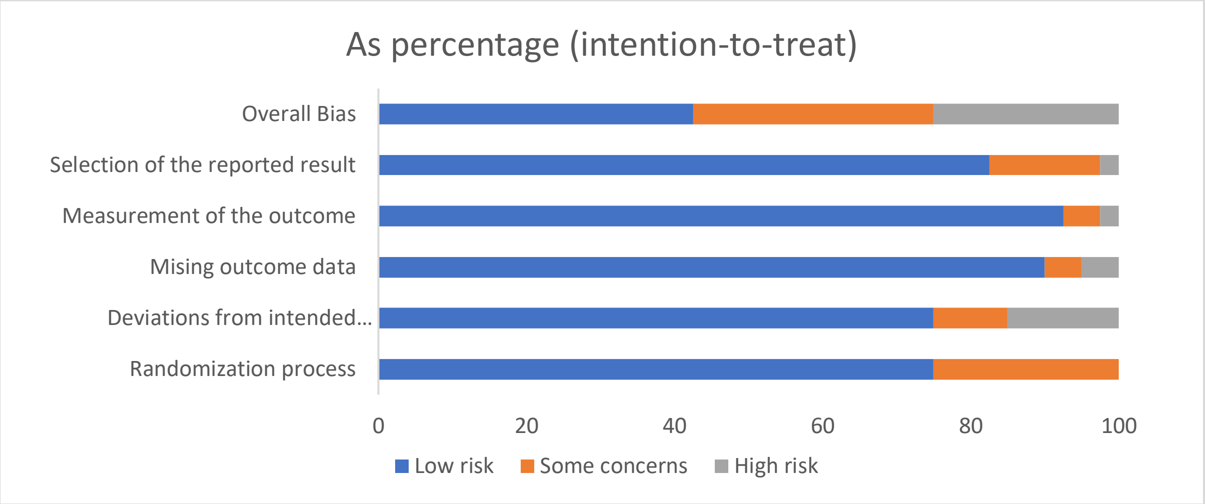

**Table S1.** Search strategy for Medline (Pubmed) and Clinicaltrials.gov

| |
|---|
| dialysis[tiab]<br>OR<br>peritoneal dialysis[tiab]<br>OR<br>hemodialysis[tiab]<br>OR<br>hemodiafiltration[tiab]<br>OR<br>hemodiafiltration[tiab]<br>OR<br>hemofiltration[tiab]<br>OR<br>Hemofiltration<br>OR<br>extracorporeal blood cleansing[tiab]<br>OR<br>Hemodialysis[tiab]<br>OR<br>Renal Dialysis[mh]<br>OR<br>Renal replacement[tiab]<br>OR<br>end stage kidney[tiab]<br>OR<br>end stage renal[tiab]<br>OR<br>stage 5 kidney[tiab]<br>OR<br>stage 5 renal[tiab] |

**Table S2.** Search strategy for Recoll (Full text search)

| |
|---|
| phase ii/iii[tiab]<br>OR<br>treatment switching[tiab]<br>OR<br>biomarker adaptive[tiab]<br>OR<br>biomarker adaptive design[tiab]<br>OR<br>biomarker adjusted[tiab]<br>OR<br>adaptive hypothesis[tiab]<br>OR<br>adaptive dose-finding[tiab]<br>OR<br>pick-the winner[tiab]<br>OR<br>drop-the-loser[tiab]<br>OR<br>sample size re-estimation[tiab]<br>OR<br>re-estimations[tiab]<br>OR<br>adaptive randomization[tiab]<br>OR<br>group sequential[tiab]<br>OR<br>adaptive seamless[tiab]<br>OR<br>adaptive design[tiab]<br>OR<br>Interim monitoring[tiab]<br>OR<br>Bayesian adaptive[tiab]<br>OR<br>Flexible design[tiab]<br>OR<br>Adaptive trial[tiab]<br>OR<br>play-the-winner[tiab]<br>OR<br>adaptive method[tiab]<br>OR<br>(adaptive[All Fields] AND dose[All Fields] AND adjusting[All Fields])<br>OR<br>response adaptive[All Fields]<br>OR<br>adaptive allocation[All Fields]<br>OR<br>adaptive signature design[tiab]<br>OR<br>treatment adaptive[tiab]<br>OR<br>covariate adaptive[tiab]<br>OR<br>sample size adjustment[tiab]. |

1 **Table S3.** Characteristics of the trials

| Study Characteristic | Categories | Description |
|---|---|---|
| Nature of Adaptive Design | GSD/SSR/DS/DE/Seamless/Interim Analysis | The type of adaptive design used in the trial. |
| Stopping Rule | Futility/Efficacy/Two sided/ N/A | If a stopping rule was used, what was the nature of the stopping rule. |
| Year of study completion | None | The year of study completion. |
| Population under study | None | A description of the population studied e.g. patients with diabetes. |
| Chronicity of KRT | Acute Kidney Injury (AKI) / kidney failure | A category for the chronicity of Kidney Replacement Therapy (KRT), either Acute Kidney Injury (AKI) or kidney failure. |
| Intervention | None | A free text description of the intervention. |
| Nature of the intervention | Medication/Medical Device/Dialysis Parameter | A category for the nature of the intervention. |
| Primary Outcome | None | A description of the primary outcome of the trial. |
| Type of primary outcome | Continuous or dichotomous | A categorial variable for the type of primary outcome variable. |
| Nature of primary outcome | Surrogate, patient-centred or hard clinical | A categorial variable for the nature of primary outcome variable either surrogate, patient-centred or hard clinical. |
| Dialysis Modality | Hemodialysis, peritoneal dialysis, hemodiafiltration or Hemofiltration | A categorial variable for the dialysis modality. |
| Sample Size of Study | None | The number of participants in the study. |
| The country of the lead investigator. | None | The country of the lead investigator. |
| The funder of the study | Public/Private | A categorial variable for source of funding for the study. |
| Study Phase | Phase II/Phase III/Combined Phase II/III | A categorial variable for study phase. |

2

**Table S4**. Risk of Bias Assessment

| Study | Author | Experimental | Comparator | Randomization process | Deviations from intended interventions | Missing outcome data | Measurement of the outcome | Selection of the reported result | Overall Bias |
|---|---|---|---|---|---|---|---|---|---|
| FENO HSR (34) | Bove et al | Fenoldopam infusion | Placebo (saline) | Low | Low | Low | Low | Low | Low |
| DAC (52) | Dember et al | Clopidogrel | Placebo | Low | Low | Low | Low | Low | Low |
| FAVOURED (67,68) | Irish et al, Viecelli et al | Fish Oil Supplementation and Aspirin Use | Placebo | Low | Low | Low | Low | Low | Low |
| Kwiatkowski et al (76) | Kwiatkowski et al | Peritoneal Dialysis | Furosemide | Low | Low | Low | Low | Low | Low |
| IVOIRE (33) | Joannes-Boyau et al | High-volume Hemofiltration | Standard-volume Hemofiltration | Some concerns | Some concerns | Low | Low | Low | Some concerns |
| CULPRIT-SHOCK (42,43) | Thiele et al | Culprit-lesion-only PCI | Immediate multivessel PCI | Low | High | Low | Low | Low | High |
| COACT (78,79) | Lemkes et al | Immediate coronary angiography | Delayed coronary angiography | Low | High | Low | Low | Low | High |
| LEVO-CTS (41,96) | Mehta et al | Levosimendan | Placebo | Low | Low | Low | Low | Low | Low |
| FRESH (80) | Douglas et al | Fluid Response Evaluation | Usual Care | Low | High | High | Low | Low | High |
| ATN (31,97) | Sharma et al | Intensive RRT | Less Intensive RRT | Low | High | Low | Low | Some concerns | High |
| IDPN-Trial (84) | Marsen et al | Intradialytic parenteral nutrition | standardized nutritional counseling | Low | Low | Low | Low | High | High |

| | | | | | | | | | |
|---|---|---|---|---|---|---|---|---|---|
| Chapman et al (51) | Chapman et al | Topical Recombinant Human Thrombin | Bovine Thrombin | Low | Low | Low | Low | Some concerns | Some concerns |
| Ejaz et al (32) | Ejaz et al | Nesiritide | Placebo | Low | Some concerns | Low | Low | Some concerns | Some concerns |
| Hemodiafe (72) | Vinsonneau | Continuous venovenous hemodiafiltration | Intermittent Hemodialysis | Low | High | Low | Low | Some concerns | High |
| ACCORD (56) | Ismail-Beigi et al | Intensive glycemic therapy with a target HbA1c of <6.0% | standard therapy with a target of 7-7.9% | Low | Low | Low | Low | Low | Low |
| HONEYPOT (61,62) | Johnson et al | Antibacterial honey | standard exit-site care | Low | High | Low | Some concerns | Low | High |
| Acker et al (30) | Acker et al | Thyroxine | Placebo | Some concerns | Low | Low | Low | Some concerns | Some concerns |
| Besarab et al (49) | Besarab et al | Normal Hematocrit Values | Low Hematocrit Values | Some concerns | Low | Some concerns | Low | Some concerns | Some concerns |
| DAC (53) | Dixon et al | Dipyridamole plus aspirin | Placebo | Low | Low | Low | Low | Low | Low |
| HALT-PKD (63) | Torres et al | Angiotensin Blockade | Placebo | Low | Low | Low | Low | Low | High |
| AKIKI (37,38) | Gaudry et al, Weisbord et al | Early phase RRT | Delayed phase RRT | Low | Low | Low | Low | Low | Low |
| PRESERVE (44) | Weisbord et al | Sodium bicarbonate | Normal saline and acetylcysteine | Low | Low | Low | Low | Low | Low |
| CREDENCE (69) | Perkovic et al | Canagliflozin | Placebo | Low | Low | Low | Low | Low | Low |

| | | | | | | | | | |
|---|---|---|---|---|---|---|---|---|---|
| DECLARE “TIMI 58 (70) | Wiviott et al | Dapagliflozin | Placebo | Low | Low | Low | Low | Low | Low |
| KALM-1 (87) | Fishbane | Difelikefalin | Placebo | Low | Low | Low | Low | Low | Low |
| Knoll et al (64,65) | Knoll et al | Ramipril | Placebo | Some concerns | Low | Low | Low | Low | Some concerns |
| Schanz et al (46) | Schanz et al | Renal consult | Usual care | Low | Low | Low | Low | Low | Low |
| CHART (85,86) | Kammerer et al | Hydroxyethyl starch | 5% albumin | Low | Some concerns | Some concerns | Low | Low | Some concerns |
| STOP-AKI (89,90) | Pickkers et al, Peters et al | Recombinant alkaline phosphatase 0.4mg/kg | recombinant alkaline phosphatase 1.6mg/kg or placebo | Low | Low | Low | Low | Low | Low |
| RICH (47,48) | Zarbock et al, Meersch et al | Regional citrate anticoagulatio n | Systemic heparin | Low | Some concerns | Low | Low | Low | Some concerns |
| CONTRAST (59,60) | Grooteman et al, The contrast group et al | Online hemodiafiltrati on | Low flux Hemofiltratio n | Low | Low | Low | Low | Low | Low |
| ELAIN-Trial (39,40) | Zarbock et al | Early RRT | Delayed RRT | Some concerns | Low | Low | Low | Low | Some concerns |
| SCD (74) | Tumlin et al | Selective cytopheretic device and CVVHD | CVVHD | Low | Low | High | Low | Low | High |
| Kratochwill et al (83) | Kratochwill et al | Alagn | Glucose based pdf | Low | Low | Low | Low | Low | Low |
| Himmelfarb et al (91) | Himmelfarb et al | THR 184 | Placebo | Some concerns | Low | Low | Low | Low | Some concerns |
| OPPORTUNIT Y (57,58) | Kopple et al | Recombinant hGH | Placebo | Some concerns | Low | Low | Low | Low | Some concerns |
| PREDICT (81,82) | Hayashi et al, Imai et al | High-hemoglobin | Low-hemoglobin | Low | Low | Low | High | Low | High |

| | | | | | | | | | |
|---|---|---|---|---|---|---|---|---|---|
| Riley et al (73) | Riley et al. | Continue CPD | Discontinue CPD | Some concerns | Low | Low | Some concerns | Low | Some concerns |
| AURORA (54,55) | Fellström et al | Rosuvastatin | Placebo | Some concerns | Low | Low | Low | Low | Some concerns |
| HEROICS (36) | Combes et al | Early high volume Hemofiltration | Standard care | Some concerns | Low | Low | Low | Low | Low |
